# Antinociceptive Action of Isolated Mitragynine from Mitragyna Speciosa through Activation of Opioid Receptor System

**DOI:** 10.3390/ijms130911427

**Published:** 2012-09-12

**Authors:** Abdul Rahman Shamima, Sharida Fakurazi, Mohamad Taufik Hidayat, Ithnin Hairuszah, Mohamad Aris Mohd Moklas, Palanisamy Arulselvan

**Affiliations:** 1 Faculty of Pharmacy, Cyberjaya University College of Medical Sciences, 63000 Cyberjaya, Selangor, Malaysia; E-Mail: alfaqirah_msc@yahoo.com; 2 Faculty of Medicine and Health Sciences, Department of Human Anatomy, Universiti Putra Malaysia, 43400 UPM Serdang, Selangor, Malaysia; E-Mails: taufik@medic.upm.edu.my (M.T.H.); aris@medic.upm.edu.my (M.A.M.M.); 3 Institute of Bioscience, Laboratory of Vaccines and Immunotherapeutics, Universiti Putra Malaysia, 43400 UPM Serdang, Selangor, Malaysia; E-Mail: arulbio@gmail.com; 4 Faculty of Medicine and Health Sciences, Department of Pathology, Universiti Putra Malaysia, 43400 UPM Serdang, Selangor, Malaysia; E-Mail: hairusza@medic.upm.edu.my

**Keywords:** antinociceptive, cannabinoid, *Mitragyna speciosa*, mitragynine, opioid

## Abstract

Cannabinoids and opioids systems share numerous pharmacological properties and antinociception is one of them. Previous findings have shown that mitragynine (MG), a major indole alkaloid found in *Mitragyna speciosa* (MS) can exert its antinociceptive effects through the opioids system. In the present study, the action of MG was investigated as the antinociceptive agent acting on Cannabinoid receptor type 1 (CB1) and effects on the opioids receptor. The latency time was recorded until the mice showed pain responses such as shaking, licking or jumping and the duration of latency was measured for 2 h at every 15 min interval by hot plate analysis. To investigate the beneficial effects of MG as antinociceptive agent, it was administered intraperitoneally 15 min prior to pain induction with a single dosage (3, 10, 15, 30, and 35 mg/kg b.wt). In this investigation, 35 mg/kg of MG showed significant increase in the latency time and this dosage was used in the antagonist receptor study. The treated groups were administered with AM251 (cannabinoid receptor-1 antagonist), naloxone (non-selective opioid antagonist), naltrindole (δ-opioid antagonist) naloxonazine (μ_1_-receptor antagonist) and norbinaltorpimine (κ-opioid antagonist) respectively, prior to administration of MG (35 mg/kg). The results showed that the antinociceptive effect of MG was not antagonized by AM251; naloxone and naltrindole were effectively blocked; and norbinaltorpimine partially blocked the antinociceptive effect of MG. Naloxonazine did inhibit the effect of MG, but it was not statistically significant. These results demonstrate that CB1 does not directly have a role in the antinociceptive action of MG where the effect was observed with the activation of opioid receptor.

## 1. Introduction

Cannabinoids have been shown to exert a broad variety of pharmacological actions, including central and peripheral effects, through receptor-mediated mechanisms [1]. The existence of an endogenous cannabinoid system, comprising cannabinoid receptor type 1 (CB1) and cannabinoid receptor type 2 (CB2) receptor subtypes together with their signaling pathways and endogenous ligands, is now well recognized [2]. The CB1 consists of 472 amino acids in human and it is expressed abundantly in the nervous system and various peripheral tissues. The CB1 receptor is assumed to be involved in the regulation of cognition, memory and motor activity [3] as well as being involved in pain management [4,5]. It has been proven that this receptor can form homodimeric and/or heterodimeric complexes with mu opioid receptor and/or dopamine D_2_ receptor respectively [6]. Antinociceptive and anti-hyperalgesic effects of cannabinoids were proved by various animal pain models [7,8]. The antinociceptive activities of cannabinoids are believed to be exerted via cannabinoid receptors (subtypes CB1 and CB2) located on brain cells and spinal cord [9].

The study of CB1 in the involvement of pain management started with the finding that *Δ*-9-tetrahydrolcannabinol (THC), the major psychoactive constituent of marijuana, enhances the potency of opioids such as morphine in animal models [10]. The first evidence of the antinociceptive effects of THC without behavioral adverse effects was published in 1994, when Smith and colleagues demonstrated that a kappa opioid receptor antagonist, norbinaltorphimine (norBNI), blocked only the antinociceptive effects of THC in rodents without resulting on hypothermia, hypo-activity or catalepsy [11,12].

Meanwhile, opioids systems have been established in treating pain. Opioids exert their antinociceptive actions from supraspinal, spinal and peripheral sites, providing a multitude of areas that may be targeted in the treatment of pain. It is well-known that the opioids exert their effects via a system of highly selective receptors and also the involvement of endogenous opioids. Opioid receptors which are comprised of three receptor subtypes (mu, kappa and delta) belong to the family of G protein-coupled receptor [13]. When the opioid is bound to the receptor, the associated G protein becomes “activated”. The activation of the receptor decreases activity of adenylyl cyclase, resulting in a decreased production of cyclic adenosine monophosphate (cAMP). This leads to an increase in the efflux of K^+^ and cellular hyperpolarization and a decrease in the influx of Ca^2+^ and lower intracellular concentrations of free Ca^2+^. The overall consequences are a decrease in the neuronal release of neurotransmitters [14] and results in analgesia [13].

Nowadays, various natural product-derived medicines have been used as alternative therapy for pain. Due to their natural constituents and high availability, natural herbs obtained from traditional natural sources are believed to provide less untoward effect profiles which may offer greater effectiveness compared to synthetic drugs available in the pharmaceutical market. The leaves of *Mitragyna speciosa* (MS) (kratom in Thai; ketum in Malaysia) have been used in Thailand and Malaysia for the opium-like effect [15,16]. Indole alkaloids, mitragynine (MG) was obtained as the major alkaloid in MS [17]. Previous studies have indicated that MG plays a major role as antinociceptive agent and acts via opioid receptors [18,19].

Various reports showed that drug addicts use the leaves as an alternative to manage drug withdrawal symptoms in Malaysia and Thailand [20,21]. Methanolic extract of *Mitragyna speciosa* possesses strong anti-inflammatory properties in experimental animals [16]. Isolated MG was reported to be comparable to codeine as an alternative analgesic and that MG is an effective opioid receptor agonist. Antinociceptive activity of MG was involved through descending noradrenergic and serotogenic systems in the tail-pinch test, but seratogenic systems were mainly involved in the hot-plate test. In *in vitro* studies, MG showed strong suppressed effect on electrically stimulated contraction of isolated guinea-pig ileum through the opioid receptor [22]. Based on these findings, MG acts through the opioid receptor system, however although various studies have been reported on the effect of MG on the opioid receptor system, there has been no systematic investigation studying the possible analgesic effect of MG on the CB1 receptor system.

Therefore, the present study has investigated whether MG exerts its antinociceptive effects through the activation of cannabinoid CB1 receptor. The antinociceptive action of MG isolated from Malaysia grown sources was confirmed together with its effective dose (ED_50_). In addition, the antinociceptive effect of MG on the cannabinoid CB1 receptor was investigated and its action on various opioid receptors.

## 2. Results and Discussion

### 2.1. Results

#### 2.1.1. Isolation of Mitragynine from *Mitragyna Speciosa* Leaves

Mitragynine is the major active compound found to be the primary active alkaloid compound and is believed to be the major contributor of various biological properties. This compound was isolated from MS and confirmed through column chromatography (CC), thin layer chromatography (TLC) and also nuclear magnetic resonance (NMR) analysis. The *Rf* value of MG from the authentic sample was 60.5. Vials with one spot with *Rf* value of 60.5 was evaluated for NMR. Overall, the yield of isolated MG was 0.871 g from the total one kilogram of MS dried leaves. From 0.871 g of MG isolated, only one vial weighted 0.4956 g was used in this present study. The isolated compound was confirmed with authentic sample by TLC with suitable solvent system, ethylacetate: hexane (7:3). Further this compound was confirmed through ^1^H-NMR, ^13^C-NMR spectral studies.

**Figure 1:**
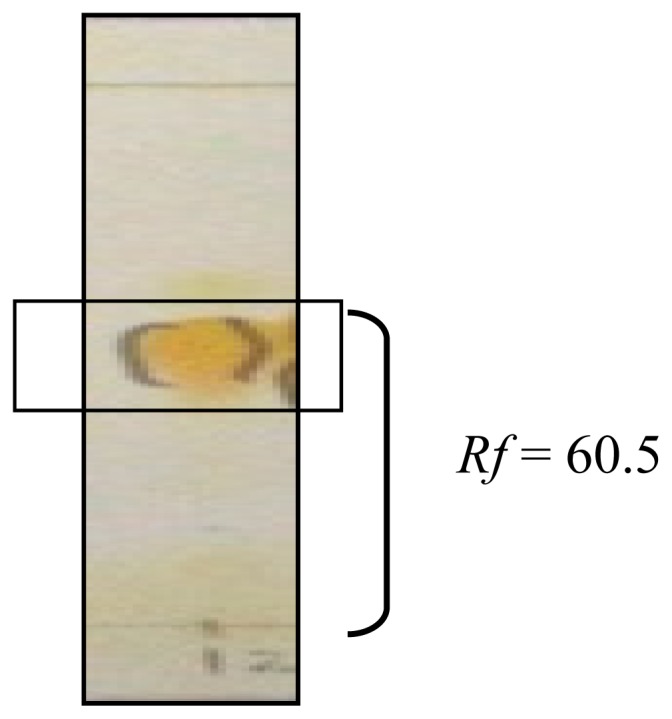
Thin layer chromatography (TLC) plate results of mitragynine (MG) using Dragendorff’s reagent and viewed with UV light microscopy.

**Figure 2:**
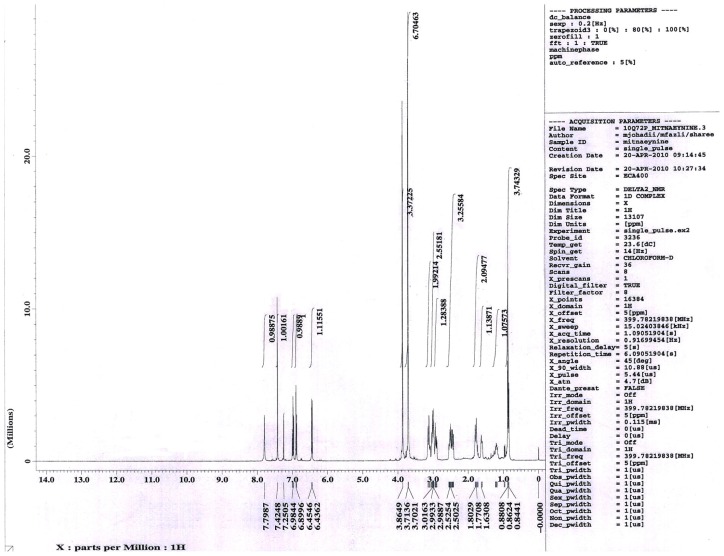
^1^H NMR chromatogram of mitragynine from *Mitragyna speciosa* leaves in deuterium chloroform.

**Figure 3:**
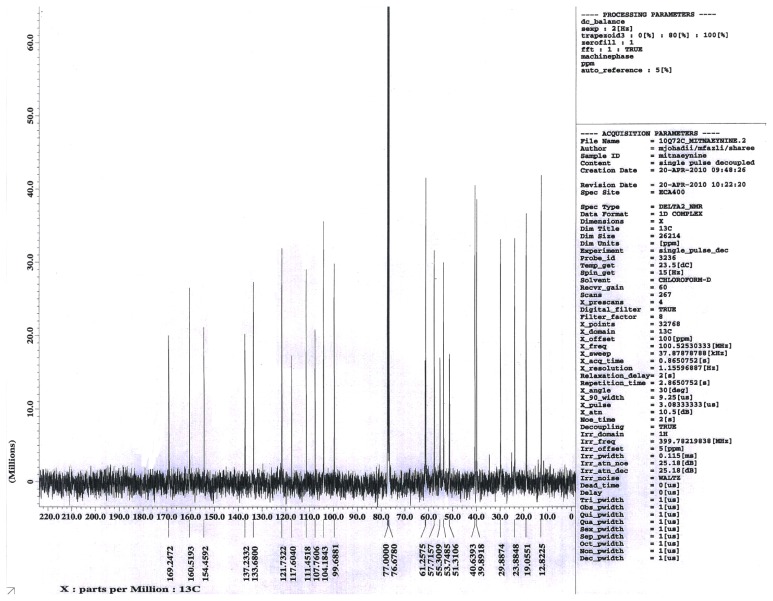
^13^C NMR chromatogram of mitragynine from *Mitragyna speciosa* leaves in deuterium chloroform.

^13^CNMR: 169.2, 160.5, 154.4, 137.2, 133.6, 121.7, 117.6, 111.4, 107.7, 104.1, 99.6, 61.2, 57.7, 55.3, 53.7, 51.3, 40.6, 39.8, 29.8, 23.8, 19.0, 12.8. The molecular formula of the isolated compound was identified as C_23_H_30_N_2_O_4_. The results of the TLC, ^1^H-NMR and ^13^ C-NMR spectra are shown in Figures 1–3.

The NMR data of isolated compound, mitragynine: ^1^H NMR: 7.79 (1H, s, –NH), 7.42–6.45 (3H, m, Ar-H), 6.43 (1H, s, =CH), 3.86 (3H, s, –OCH_3_), 3.71 (3H, s, –OCH_3_), 3.70 (3H, s, –OCH_3_), 3.01–2.99 (4H, m, –N–CH_2_), 2.98 (1H, s, –CH), 2.52–2.51 (2H, m, –CH_2_), 2.50 (1H, s, –CH), 1.63 (1H, s, –CH), 1.80–1.77 (2H, m, CH_2_), 1.53–1.33 (2H, m, –CH_2_), 0.862 (3H, t, *J* = 7.5 Hz).

#### 2.1.2. Effects of Mitragynine on Hot-Plate Test (HPT) and the Determination of Effective Dose 50 (ED_50_)

**Figure 4:**
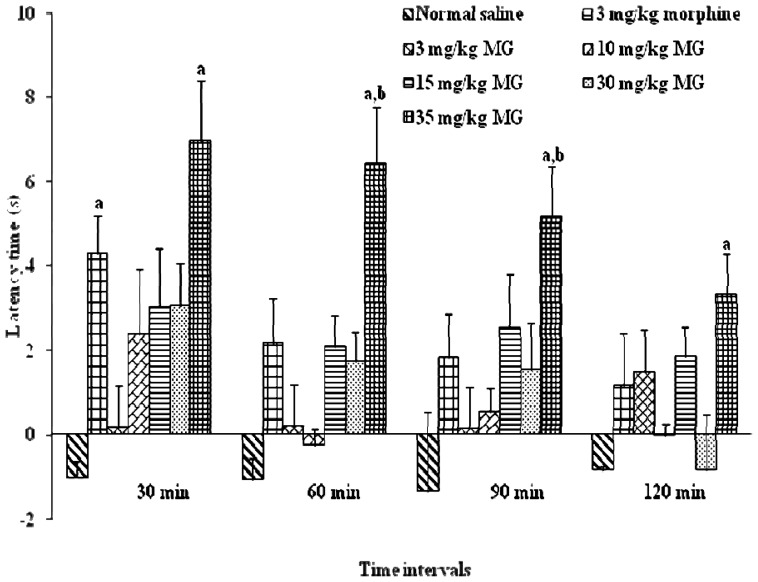
The effects of mitragynine (3 mg/kg, 10 mg/kg, 15 mg/kg, 30 mg/kg and 35 mg/kg), morphine and control on latency time in hot-plate test (HPT) for various time intervals. Mitragynine was administered 15 min before the HPT intraperitoneally (i.p). Each column represents the mean ± SEM of latency time (s) for group of eight animals in each group. Values are statistically significant at *p* < 0.05. ^a^ 35 mg/kg b.wt of MG treated groups were compared to normal saline (vehicle control) group; ^b^ 35 mg/kg b.wt of MG treated groups were compared to 3 mg/kg b.wt morphine group.

**Figure 5:**
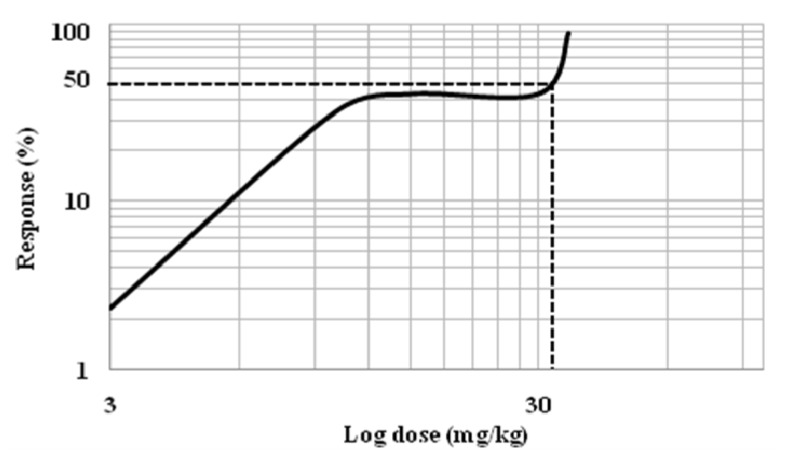
The determination of effective dose 50 (ED_50_) of mitragynine. Dose-response curves of antinociceptive effect of mitragynine in a hot plate test. The effect of mitragynine was assessed every 30 min during the total experiment period (120 min).

MG (3–35mg/kg, i.p) was found to induce dose-dependent antinociceptive response in the hot plate test. In Figure 4, the administration of 15 mg/kg of MG increased the latency period. The dose dependent effect was observed at all-time points. The best antinociceptive effect was observed with concentration 35 mg/kg of MG that resulted in a significant increase (*p* < 0.05) in latency time when compared to vehicle and morphine groups. Dose-response curve was generated from each dosage to determine the effective dose 50 (ED_50_) of MG (Figure 5). MG dose ranging from 3 to 60 mg/kg b.wt was tested for antinociceptive effects in experimental system. Findings from the dose response curve, mitragynine was showed significant antinociceptive effect and this effect of MG was showed in dose dependent manner. However, the antinociceptive effect of MG was marginally reduced at 40 mg/kg and 60 mg/kg (data not shown).

#### 2.1.3. The determination of the Effect of Mitragynine Following the Administration of Antagonists

##### 2.1.3.1. The Determination of the Effect of Mitragynine Following the Administration of CB1 Antagonists

**Figure 6:**
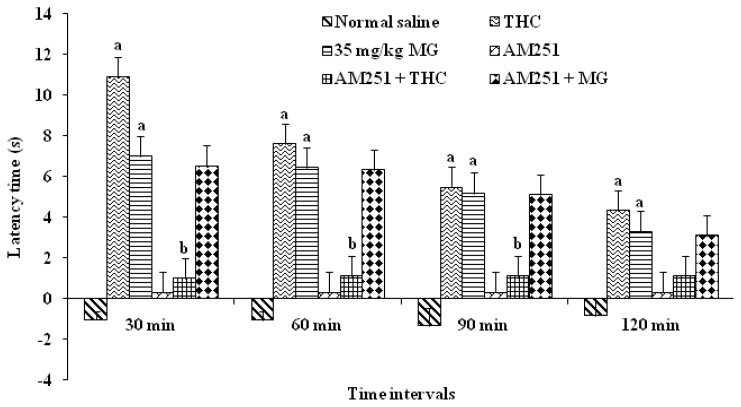
Effects of cannabinoid 1 receptor (CB1) antagonist AM251 on mitragynine-induced antinociception (35 mg/kg). Each column represents the mean ± SEM of latency time (s) of six animals in each group. Values are statistically significant at ^a^ *p* < 0.05 compared to normal saline (vehicle control) and ^b^ *p* < 0.05 compared to THC alone group.

In order to determine which receptors were involved in antinociceptive effects of MG, we utilized receptor antagonists directed against the CB1. Receptor antagonist was administered prior to the agonist, as described in the experimental section to confirm that each antagonist blocked the respective receptor involved. As positive control, *Δ*-9-tetrahydrocannabinol (THC) was used. The result showed that THC increases the latency period (*p* < 0.05) which showed antinociceptive effects. The latency time was increased significantly, up to 60 min compared to vehicle treated groups. After 60 min, the effect decreased and was not significant when compared to the vehicle group. The MG alone group also showed a significant increase in the latency period compared to vehicle group and the significant antinociceptive effect was seen throughout the period of 120 min. The antinociceptive effect of THC was blocked by AM251. This was proved when the result showed that the latency time was significantly decreased at 30 min when compared to THC alone group (*p* < 0.05). However, when MG was given following prior administration of AM251, the latency time was prolonged and it was not statistically significant when compared to group with MG alone (Figure 6). These results suggested that MG did not exert the antinociceptive action through the CB1 receptor.

##### 2.1.3.2. The Determination of the Effect of Mitragynine Following the Administration of Opioid Receptor Antagonists

**Figure 7:**
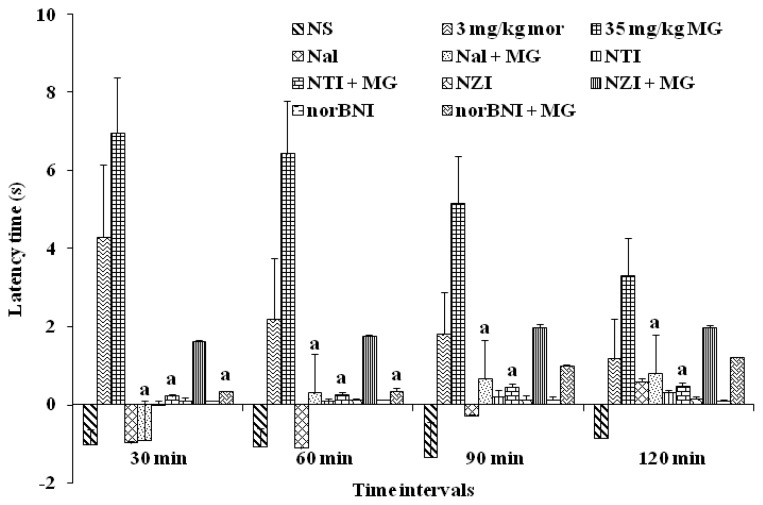
Effects of μ-opioid antagonist naloxone, δ-opioid antagonist naltrindole, μ_1_-opioid antagonist naloxonazine and κ-opioid antagonist norBNI on mitragynine-induced antinociception (35 mg/kg). Each column represents the mean ± SEM of latency time (s) for group of eight animals in each group. Values are statistically significant at *p* < 0.05. ^a^ *p* < 0.05 compared to mitragynine (35 mg/kg) alone group.

Naloxone (Nal), a non-selective opioid receptor antagonist, completely reversed the antinociceptive effects of MG when it showed a significant decrease in the latency time compared to MG alone. The same result was observed by naltrindole (NTI), a δ-receptor antagonist. Naloxonazine (NZI), a μ_1_-receptor antagonist did reduce the anticociceptive effect of MG, but it is not statistically significant. This indicates that MG may not only act specifically on μ_1_-receptor. However, norbinaltorpimine (norBNI) partially blocked the effect of MG and significantly decreased the latency time when compared with MG alone group from 30 min to 60 min, but not up to 120 min time. These findings indicate that MG may partially act via κ-receptor (Figure 7).

### 2.2. Discussion

Mitragynine isolated from MS is known as an indole alkaloid. Alkaloids are defined as basic nitrogenous plant products, mostly optically active and possessing nitrogen heterocycles as their structural units, with a pronounced physiological action [23]. Isolation and purification of an alkaloid from a plant is not a simple process because generally they contain a complex mixture of several alkaloids. In addition, products like glycosides, organic acids, and others present in plants may complicate this isolation further. Thus, the isolation of a pure alkaloid sometimes may become an extremely laborious procedure [23].

In the present study, MG which has been identified according to thin layer chromatography (TLC) method was then analyzed using ^1^H-NMR and ^13^C-NMR to further confirm the purity and authenticity of MG. The standard ^1^H-NMR and ^13^C-NMR is able to identify MG used in this study as described in previous reports by Chittakarn *et al*. [24], Kumarnsit *et al*. [25,26] and Houghton *et al*. [27]. From the ^1^H-NMR and ^13^ C-NMR spectra, a total of 23 carbon and 30 hydrogen resonances and the molecular formula, C_23_H_30_N_2_O_4_ was derived (Figures 1–3).

The antinociceptive activities are determined by exposing animals to potentially painful stimuli such as heat or electric shock and measuring either the time it takes them to respond to the stimuli or the intensity at which they respond. If the time taken by the animal to respond to the stimulus is prolonged following compound or drug administration, then it is presumed that the compound and/or drug have altered the perception to painful stimulus. Increase in latency time until the occurrence of nociceptive responses (licking, shaking legs or jumping) reflects that there is an alteration to painful stimulus, and that the tested compounds have antinociceptive properties. Yaksh [28] indicated that the hot-plate test (HPT) was also used to study the possible involvement of supraspinal receptors whereas tail-flick test was used to investigate the involvement of spinal opioid receptors (Figure 4).

In this study, antinociceptive effect of MG was evidenced by the increase of latency time in the HPT. The increase of latency time is dose dependent with significant increase of dosage up to 35mg/kg. The effect of MG as antinociceptive agent was decreased in much higher dosage up to 60 mg/kg (data not shown). Interestingly, the increase of latency period was higher than that of morphine group. The morphine group only showed an increase in the latency period at 30 min and it is significant when compared to control groups. This finding was in line with the previous findings by Matsumoto *et al*. [29] which found that MG possesses an antinociceptive activity when given i.p and i.c.v in tail-pinch and hot-plate test.

In the present investigation, the effect of MG in HPT was maximal between 30 min–60 min which is slightly slower than the previous study by Matsumoto *et al*. [30] which indicates maximal antinociceptive effects at 15 min–45 min after injection. The differences may be due to the sources of MG, which in this study was from the Malaysian Peninsular whereas the study by the Japanese group isolated MG from a source in Thailand. It was revealed by Takayama *et al.* [17] and Ikram [31] that MG isolated from plants in Thailand consists of a stronger MG in biological activity and percentage.

Selective antagonists were employed in order to clarify the involvement of the receptor subtypes in the antinociceptive effect of MG. CB1 receptor is proved to have a role in pain management. The study of cannabinoids as analgesics stems from findings that compounds like *Δ*-9-tetrahydrocannabinol (*Δ*-9-THC), antinociception can produce antinociception without respiratory depression associated with opioid analgesics [32]. CB1 receptor antagonist which is AM251 was used in this study.

From the result, *Δ*-9-THC was completely blocked by the cannabinoid CB1 receptor antagonist AM251, as expected. This confirms that *Δ*-9-THC exerts its antinociceptive effect via the CB1 receptor in the HPT. However, the effect of MG was not blocked by AM251 antagonist. Previous findings by Rios *et al*. [33] have suggested that there is a formation of heteromeric receptor complexes between opioid specifically μ-opioid receptor and CB1 receptor which contribute to functional interaction between these two classes of agonist which may lead to antinociceptive action. In this study, it shows that antinociceptive action of MG does not directly involve the cannabinoid CB1 receptor system (Figure 6).

To investigate which opioid receptor subtypes are involved in the pharmacological effects of MG, further investigations using selective opioid antagonist have been carried out with a 35 mg/kg dosage of MG. The antinociceptive effect of i.p injection of MG was completely blocked by naloxone given i.p, a non-selective opioid receptor antagonist, indicating that opioid receptor systems are involved in the action of MG. These findings are consistent with the findings of Matsumoto *et al*. [30] who reported that i.p and i.c.v administration of naloxone blocked the antinociceptive effects of MG but it differs with the findings reported by Macko *et al*. [34] who found that i.p administration of naloxone did not block the antinociceptive actions of MG (Figure 7).

This study also supported the findings that besides μ-opioid receptors, δ-receptors are also involved in the mechanism of action of MG as antinociceptive compound. This was proved when the antinociception caused by MG was also significantly antagonized by co-administration of naltrindole, a δ receptors antagonist. With the co-administration of nalozonazine, however, a specific μ_1_-receptor antagonist, even though it did inhibit the antinociceptive action of MG, it was not statistically significant. This proved the antinociceptive effects of MG, not only through μ_1_-receptor, but through both μ-receptor subtypes.

The present findings showed significant difference between previous reports when MG blocked the effect of norbinaltorphimine, a selective κ-opioid receptor antagonist for 60 min, whereas in the case of Thongpradichote, [35] reported that MG blocked the effect of norbinaltorphimine, a selective κ-opioid receptor antagonist in the tail-pinch test but not in hot plate analysis (Figure 7).

## 3. Experimental Section

### 3.1. Isolation of Mitragynine from *Mitragyna Speciosa* Leaves

*Mitragyna speciosa* leaves were collected from natural sources around Peninsular Malaysia and authenticated by a botanist from the Faculty of Forestry, Universiti Putra Malaysia. A voucher specimen (ATS: 001) has been kept at the herbarium for future reference. Mitragynine was isolated from leaves of *Mitragyna speciosa* according to the methods described by Houghton and Ikram [27], Ponglux *et al*. [36] and Reanmongkol *et al*. [37] with a slight modification.

**Figure 8:**
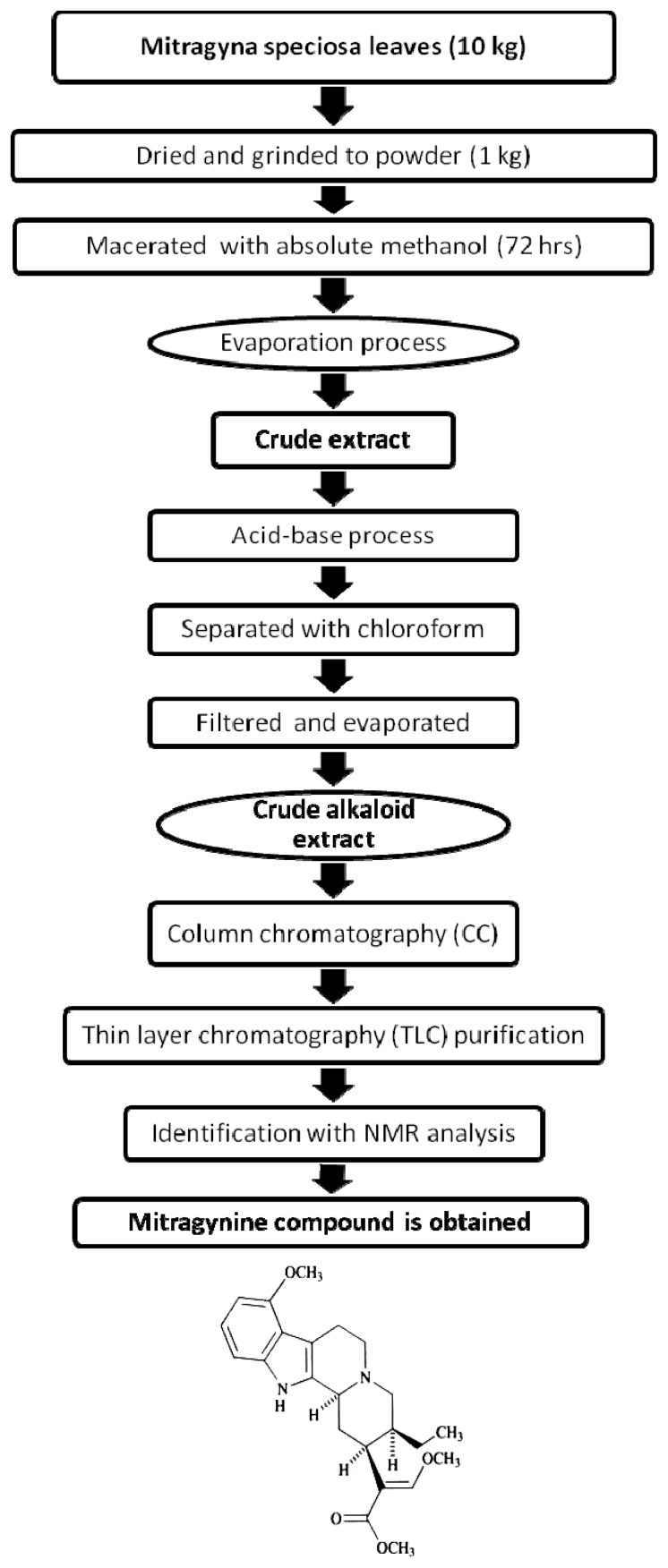
Schematic representation of isolation of mitragynine from *Mitragyna speciosa.*

One kilogram leaves of MS was cleaned and dried with constant temperature at 45 °C overnight before grinded into powder form. It was then macerated with absolute methanol for 72 h. The mixture was filtered to remove filtrate from insoluble particles to obtain the methanol extract. Extract was evaporated using rotary evaporator (Eyela, Japan) at a temperature below 55 °C. The methanol extract was added with 5% sulphuric acid and extensively stirred overnight. The mixture was filtered and a clear yellow solution of acidic filtrate was obtained. The acidic filtrate was then mixed with sodium carbonate and stirred until it turned to dark grey with pH 11 to become basic filtrate. To separate the alkaloids from the basic crude extract, chloroform was added to mixture in separating funnel. Component of three layers were produced which were the aqueous, salt and chloroform layer. The chloroform layer was then separated and filtered out from the mixture. The chloroform fraction was mixed with sodium sulfate anhydrous and evaporated to yield 0.73% (*w*/*w*) of crude extract. The major alkaloid isolated by silica gel chromatography and thin layer chromatography eluting with diethyl ether was identified as MG with standard nuclear magnetic resonance method (^1^H NMR, ^13^C NMR). Over all, the yield of MG was approximately 0.087% (*w*/*w*) of fresh leaves (Figure 8).

### 3.2. Animals

Male ICR mice weighing 25 g–35 g were housed in Animal Center, Faculty of Medicine and Health Sciences (FMHS), Universiti Putra Malaysia, Malaysia. Animals were divided into different experimental groups (*n* = 8) in a temperature-controlled room. They were maintained under standard laboratory conditions with natural dark and light cycle and fed with standard commercial food pellets and water *ad libitum*. Animals were acclimatized for at least 7 days to adapt to the laboratory prior to experiment. All animal procedures were approved (UPM/FPSK/PADS/BR-UUH/00307) by Institutional Animal Care and Use Committee (IACUC), FMHS, Universiti Putra Malaysia (UPM), Malaysia.

### 3.3. Drugs

The drugs used in this study were morphine hydrochloride (Sigma-Aldrich, USA), polyoxyetheylene sorbitan monooleate (Tween 80, Sigma-Aldrich, USA), selective opioid antagonist naloxone hydrochloride, naloxonazine, norbinaltorphimine, naltrindole (Lipomed Inc., USA), while the selective cannabinoid type 1 antagonist was; 1-(2,4-diclorophenyl)-5-(4-iodophenyl)-4-methyl-*N*-1-piperidinyl- 1-*H*-pyrazole-3-carboxamide (AM251) (Lipomed Inc., USA). *Δ*-9-Tetrahydrocannabinol (THC) also been used (Sigma-Aldrich, USA). Mitragynine and morphine were dissolved in 2.0% (*v*/*v*) Tween 80 and normal saline (0.9% NaCl) respectively. All reagents used in the study were of analytical grade.

### 3.4. Antinociceptive Study and the Determination of Effective Dose 50 (ED_50_)

Mice were randomly assigned into one normal saline (vehicle control) group and six treatment groups with eight mice per group. The pre-drug latency was tested before used where the mice with pre-drug latency below five seconds or more than 20 s were eliminated from the study. This was to avoid any bias for this experiment since the mice with extremely short or long pre-latency measurement could result in some variances in the analysis. Experimental groups assigned to: Normal saline (Vehicle control); Morphine (3 mg/kg); Mitragynine (3 mg/kg; 10 mg/kg; 15 mg/kg; 30 mg/kg and 35 mg/kg b.wt). Administration of all drugs was performed through intraperitoneal (i.p) injection at a volume of 0.5 mL/kg b.wt. All the treatments were given 15 min before perform the hot-plate test. The latency time was measured continuously after injection for 2 h at 30 min intervals. The ED_50_ value of the compound was determined and same concentration was used for receptor antagonist study.

### 3.5. The Determination of the Effect of Mitragynine Following the Administration of Cannabinoid and Opioid Antagonists

Mice were divided into fifteen groups (*n* = 8) that were pretreated with selected cannabinoid and opioid receptor antagonist. The cannabinoid receptor 1 antagonist, AM251 (3 mg/kg) was administered intraperitoneally (i.p) 30 min before MG and *Δ*-9-tetrahydrocannabinol (*Δ*-9-THC) injection. For opioid antagonists, naloxone hydrochloride (Nal) (1 mg/kg), naloxonazine (NZI) (10 mg/kg), norbinaltorphimine (norBNI) (10 mg/kg) and naltrindole (NTI) (3 mg/kg) were administered i.p. 15 min, 24 h, 1 h, 30 min, respectively before MG (35 mg/kg) or morphine injection (3 mg/kg). At the end of the experiment period, all mice were sacrificed.

### 3.6. Hot-Plate test

In the hot-plate test, mice were placed on a stainless steel surface which was maintained at the temperature of 50 °C ± 0.2 °C (Ugo Basile, Italy, Model 7280) and the latency time was assessed. The latency time is defined as the period of time when the mice were placed in the hot surface until the occurrence of nociceptive responses such as licking, shaking or jumping was observed. The cut-off time of 50 s was used to prevent tissue damage during the experiment. 30 min prior to treatment, the nociceptive threshold was measured and the latency time had been used as the pre-drug latency for each animal. The latency time was calculated as:

Latency time (s)=time when the mice showed nociceptive responses (s)-pre drug latency (s)

### 3.7. Statistical Analysis

The results were expressed as mean ± standard error (SEM) for eight mice in each group. Comparisons between experimental and control groups were analyzed by using one-way analysis variance (ANOVA) coupled with post hoc Tukey’s HSD test. Values with *p* < 0.05 were considered to indicate statistical significance.

## 4. Conclusions

In conclusion, our results show that administration of MG is able to produce an antinociceptive effect in HPT. The mechanism of action for MG is not directly through the cannabinoid receptor-1 (CB1) system but through the supraspinal opioid receptor systems. Further molecular investigations are in progress in our laboratory to elucidate its specific mechanism (s) of action.
